# UPDG: Utilities package for data analysis of Pooled DNA GWAS

**DOI:** 10.1186/1471-2156-13-1

**Published:** 2012-01-17

**Authors:** Daniel WH Ho, Maurice KH Yap, Shea Ping Yip

**Affiliations:** 1 Centre for Myopia Research, School of Optometry, The Hong Kong Polytechnic University, Hong Kong SAR, China; 2 Department of Health Technology and Informatics, The Hong Kong Polytechnic University, Hong Kong SAR, China

## Abstract

**Background:**

Despite being a well-established strategy for cost reduction in disease gene mapping, pooled DNA association study is much less popular than the individual DNA approach. This situation is especially true for pooled DNA genomewide association study (GWAS), for which very few computer resources have been developed for its data analysis. This motivates the development of UPDG (Utilities package for data analysis of Pooled DNA GWAS).

**Results:**

UPDG represents a generalized framework for data analysis of pooled DNA GWAS with the integration of Unix/Linux shell operations, Perl programs and R scripts. With the input of raw intensity data from GWAS, UPDG performs the following tasks in a stepwise manner: raw data manipulation, correction for allelic preferential amplification, normalization, nested analysis of variance for genetic association testing, and summarization of analysis results. Detailed instructions, procedures and commands are provided in the comprehensive user manual describing the whole process from preliminary preparation of software installation to final outcome acquisition. An example dataset (input files and sample output files) is also included in the package so that users can easily familiarize themselves with the data file formats, working procedures and expected output. Therefore, UPDG is especially useful for users with some computer knowledge, but without a sophisticated programming background.

**Conclusions:**

UPDG provides a free, simple and platform-independent one-stop service to scientists working on pooled DNA GWAS data analysis, but with less advanced programming knowledge. It is our vision and mission to reduce the hindrance for performing data analysis of pooled DNA GWAS through our contribution of UPDG. More importantly, we hope to promote the popularity of pooled DNA GWAS, which is a very useful research strategy.

## Background

Over the years, many methods and algorithms have been developed for genetic association studies. With the availability of DNA microarrays and their common use in genomewide association study (GWAS), the dramatic increase in the number of markers to be handled poses a great challenge to the data analysis. Owing to the inability to analyze GWAS data manually, useful computer programs have been developed, but are mainly focused on the application for GWAS based on analysis of individual DNA samples (hereafter called individual DNA GWAS). Despite being a well-established strategy for cost reduction [1], association study based on pooled DNA is far less popular than the "individual DNA" approach. There are very few available resources for the analysis of pooled DNA GWAS data. This is especially true for pooled DNA GWAS that is conducted using the Illumina platform. The GenePool package [2] is supported for Linux environment only in its stable version although multiple environments (OSX, Windows and Unix-like) are supported in its new beta version. However, its documentation is rather brief. Another available software package is MPDA [3]. Its graphical user interface is dependent on the proprietary MATLAB computing environment while its command line version relies on the MATLAB runtime environment. Another major constraint is that MPDA can only handle data from one or two data pools. In most applications, multiple DNA pools are constructed and each tested in multiple technical replicates - a study design that cannot be properly handled by MPDA. The SNPMaP package [4] was developed solely for the Affymetrix platform. Its functionality is limited to fundamental data manipulation and does not support subsequent association testing. Recently, GPFrontend and GPGraphics [5] were developed partly using modified source codes of *gpextract *and *gpanalyze *of GenePool. GPFrontend is essentially a wrapper tool for modified *gpextract *and *gpanalyze *of GenePool while GPGraphics incorporates graphical output functionality. Besides, this package is platform-independent and has been tested in Windows and Linux environments.

In order to facilitate the application of cost-saving pooled DNA GWAS, there is a need for more freely available platform-independent computer resources that are executable under different system environments for this purpose. With more software resources freely available, users are provided with more alternatives to suit their own specific needs. This motivates the development of UPDG - utilities package for data analysis of pooled DNA GWAS (Additional file 1). This utilities package consists of Unix/Linux shell operations and Perl programs for data manipulation, R scripts for data testing and a comprehensive user manual providing the instructions and procedures for pooled DNA GWAS data analysis. Users of UPDG are provided with a free, simple and platform-independent solution to pooled DNA GWAS from manipulation of raw data to summarization of analysis results.

UPDG manipulates raw GWAS data into the required data file formats. It implements pooled allele frequency estimation methods that incorporate adjustment for allelic preferential amplification/hybridization [6,7] and normalization [8] along with the unadjusted pooled allele frequency estimation. Allelic preferential amplification/hybridization denotes the situation that equal dosage of two alleles in heterozygotes does not give equal fluorescence intensity signals in microarray-based genotyping experiments. UPDG also carries out nested analysis of variance (ANOVA) on replicates of DNA pools of subject groups [9,10]. With unformatted results generated from R, it can summarize analysis results according to user-defined threshold. Pooled allele frequency estimates are also summarized for easy comparison.

## Implementation

### Programs and interface

UPDG is a utilities package developed based mainly on Unix/Linux shell operations, Perl programming language and R programming language. Unix/Linux shell operations can be executed in Unix/Linux environment directly or in Windows environment upon the installation of Cygwin (http://www.cygwin.com/). All the Perl programs in the package can be executed in Unix/Linux environment directly or in Windows environment through the installation of ActivePerl (http://www.activestate.com/activeperl). Before executing the R scripts in UPDG, R should first be downloaded from the Comprehensive R Archive Network (CRAN) website (http://cran.r-project.org/) and installed. Alternatively, users can use the shell and batch scripts provided to execute a series of UPDG components sequentially and automatically. This can simplify the overall workflow. Through the use of UPDG, the following tasks can be made easy and automated: initial manipulation of raw intensity data, generation of pooled allele frequency data corrected for allelic preferential amplification [6,7] and normalization [8], nested ANOVA analysis for genetic association testing [9,10], and summarization of analysis results and pooled allele frequency estimates. A comprehensive user manual can be found within the UPDG package, describing the details of the procedures, data file formats and functionalities for various components of the package. A small example data set is also included in the package together with the expected output files.

### Genotyping platform and data file format

All the genotyping experiments (pooled samples and individual samples) were performed with genomic DNA using the Illumina Human610-Quad BeadChips with 620901 markers, median inter-marker spacing of 2.7kb and 100% median genomic coverage in Asians (Illumina). Despite that UPDG was tested using data generated from the Illumina platform, data from other genotyping platforms can also be handled by UPDG provided that the data are first transformed to the required data formats for the corresponding components of UPDG. Detailed data file formats can be found in the user manual of UPDG. In particular, intensity data from Affymetrix CEL files can first be extracted using such free packages as SNPMaP [4] (an R package that can process CEL files to generate raw intensity data) or using the Affymetrix power tools (APT) provided by Affymetrix. Once the required data files are prepared, data analysis can then be handled by UPDG.

## Results and Discussion

### Package overview

The UPDG utilities package is targeted to carry out data analysis for pooled DNA GWAS. Its functionalities are based on the combined efforts from several Unix/Linux shell operations, Perl programs and R scripts, as summarized in Table 1. It has integrated several most commonly used methods for correcting allelic preferential amplification [6,7] and normalization [8] along with unadjusted estimation. As advanced programming knowledge is not assumed for the users of UPDG, commands for various operations are illustrated with an example dataset. Simple and straightforward instructions together with detailed procedures are provided in the easily understandable user manual. By utilizing the functionalities of UPDG, operations are completed automatically by the components of UPDG through simple commands and with minimal user intervention. UPDG represents a generalized framework suitable for analyzing data from both Illumina and Affymetrix platforms (detailed instructions are provided in the user manual on how to convert raw data to appropriate data file format). Currently, UPDG accepts summary intensity data for individual SNPs (as output from default software for raw data generation and extraction, e.g. BeadStudio or Affymetrix Power Tools). For Affymetrix CEL files, users can convert them to raw intensity data by other freely available software package (e.g. SNPMaP [4]) or using the Affymetrix power tools (APT) provided by Affymetrix, and then to the required data file format according to the instructions of the UPDG user manual. As a whole, UPDG provides a one-stop analysis tool for pooled DNA GWAS and there is no need to use other programs or packages to complete the analysis.

**Table 1:** Summary of various components of UPDG.

UPDG component	Function
merge.pl	Combines genotype and intensity data of individual DNA GWAS from separate files
adjustment.pl	Estimates allele frequencies of markers from DNA pools and performs adjustment correcting for allelic preferential amplification with the methods based on Hoogendoorn *et al. *(2000), Meaburn *et al. *(2006) and Craig *et al. *(2005), and generates input data files for the subsequent step of nested ANOVA
QC.pl	Removes SNPs with minor allele frequencies and call rates below a user-specified threshold and generates filtered input data file for nested ANOVA
nested_ANOVA_[H/M/N/U].r	Carries out nested ANOVA in R environment
output_format.pl	Organizes results from nested ANOVA and generates a summary of markers with p values below a user-specified threshold
mean_Rx_statistics.pl	Generates summary information on estimated allele frequencies for markers

### Package execution

**Figure 1:**
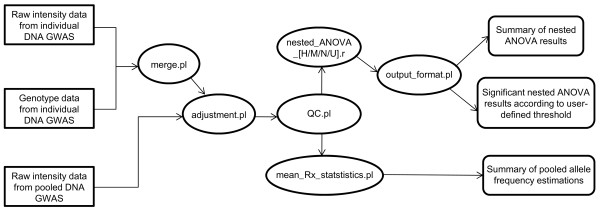
**Workflow of UPDG**.

We assume here that the data files are generated from genotyping experiments performed using the Illumina platform (e.g., Human610-Quad BeadChips). Raw fluorescence signal intensities for the two alleles of markers are extracted for both individual DNA samples and pooled DNA samples. In addition to raw fluorescence signal intensities, genotype calls are also extracted for individual DNA samples. The data extraction is carried out using Unix/Linux shell operations as illustrated in the user manual of UPDG. The raw intensity and genotype data for heterozygous individuals are combined using a Perl program (merge.pl). With these extracted data, adjustment of allelic preferential amplification by various methods, normalization and filtering for data with user-specified low minor allele frequency and low completion rate are undertaken by two Perl programs (adjustment.pl and QC.pl). With adjusted and filtered data for pooled allele frequency estimates, both summarization using another Perl program (mean_Rx_statistics.pl) and nested ANOVA using R scripts (nested_ANOVA_[H/M/N/U].r) can then be performed. Nested ANOVA assesses the differences of mean pooled allele frequencies between the case group and the control group, and hence detects the association between mean pooled allele frequency estimates and a dichotomous disease phenotype. Last but not least, the Perl program output_format.pl summarizes in a user-friendly manner the unformatted nested ANOVA results obtained using different allelic preferential amplification adjustment methods, and significant markers satisfying a user-defined threshold are also extracted. The overall workflow for UPDG is illustrated in Figure 1. Apart from executing the individual UPDG components manually by typing the commands one by one, users can also execute a series of commands through the use of shell and batch scripts provided.

### An example of Illumina Human610-Quad BeadChip data

UPDG was used to process and analyze real data obtained using the Illumina Human610-Quad BeadChips. Pooled DNA dataset consisted of 6 case pools and 6 control pools. Each pool was constructed by mixing equal amounts of 50 individual DNA samples and hence 6 case pools were created from 300 case samples and 6 control pools from 300 control samples. Each pool was tested in 3 technical replicates. Individual DNA dataset was obtained from a group of 100 cases and 100 controls. The Illumina Human610-Quad BeadChip contains 620901 markers. For pooled DNA dataset, 598821 SNPs remained for further analysis after filtering for SNPs with NCBI reference SNP (rs) numbers. Subsequent extraction of autosomal markers reduced the number of SNPs down to 582539. After further quality checking for minor allele frequency (0.01) and genotype call rate for markers (80%), there remained 581714 SNPs (adjustment based on Hoogendoorn et al. [6]), 581714 SNPs (adjustment based on Meaburn et al. [7]), 522692 SNPs (normalization based on Craig et al. [8] and adjustment based on Meaburn et al. [7]) and 581724 SNPs (unadjusted estimation). If a relatively lenient p value threshold (0.0001) was adopted for nested ANOVA analysis in at least one of the four datasets, 15 SNPs were suggestive and would be further confirmed by genotyping individual DNA samples.

To evaluate the accuracy of allele frequency estimation, 108 SNPs genotyped for DNA pools were selected on the basis of different sets of criteria and further genotyped for the 600 individual DNA samples (300 cases and 300 controls) that were used in constructing the corresponding pools. We compared the allele frequencies estimated using DNA pools and using individual DNA samples (as the reference) (Table 2). There was a general trend of over-estimation in allele frequencies for DNA pools irrespective of the correction methods (Hoogendoorn et al. [6], Meaburn et al. [7], Craig et al [8] plus Meaburn et al. [7], and unadjusted estimation). Allele frequencies were over-estimated for case pools or control pools in ~90% (range: 85.7% - 99.1%) of the SNPs tested. The differences in allele frequencies between the case and control groups were also over-estimated for DNA pools in most SNPs (~70%) examined. The most accurate estimates of allele frequencies were obtained with the correction methods based on Craig et al [8] plus Meaburn et al [7]: the mean difference was 0.0477 for the case group and 0.0352 for the control group (Table 2). On the other hand, the most accurate estimates of allele frequency differences between the case and control groups were those based on Hoogendoorn's correction method [6] or those without adjustment (mean difference being 0.0099 in both cases) (Table 2).

**Table 2:** Accuracy of allele frequency estimation for DNA pools.

Correction method^a^	Allele frequency difference (Pools - Individual samples)			Absolute values^b^		% of SNPs with over-estimated allele frequency^c^
			
	Sample group	Mean difference	SD	Mean difference	SD	
1	Case	0.0726	0.0414	0.0728	0.0411	99.1
2	Case	0.0725	0.0419	0.0725	0.0418	99.1
3	Case	0.0477	0.0435	0.0487	0.0423	94.3
4	Case	0.0690	0.0511	0.0740	0.0435	92.6
1	Control	0.0627	0.0467	0.0630	0.0463	99.1
2	Control	0.0625	0.0465	0.0626	0.0463	99.1
3	Control	0.0352	0.0512	0.0389	0.0484	85.7
4	Control	0.0591	0.0500	0.0637	0.0438	93.5
1	Case - Control	0.0099	0.0276	0.0246	0.0158	70.4
2	Case - Control	0.0100	0.0275	0.0247	0.0157	69.4
3	Case - Control	0.0125	0.0328	0.0298	0.0184	68.6
4	Case - Control	0.0099	0.0293	0.0253	0.0176	69.4

^a ^Correction methods used include those based on (1) Hoogendoorn et al [6], (2) Meaburn et al [7], (3) Craig et al [8] plus Meaburn et al [7], and (4) no adjustment.

^b ^Mean of the absolute values for the differences in allele frequencies between DNA pools and individual samples.

^c ^In total, 108 SNPs were compared for allele frequency differences as estimated for DNA pools and individual DNA samples. However, only 105 SNPs were compared for the correction method (3) (Craig et al [8] plus Meaburn et al [7]) because 3 SNPs were filtered out.

Accuracy of the correction factors for adjusting allelic preferential amplification depends on the number of heterozygotes individually tested by the same platform. The more heterozygous samples are tested individually, the more accurate the correction factors are, but the less cost-effective the DNA pooling approach becomes. As the main targets of GWAS are common variants (usually at least a minor allele frequency of 5%), we expect to find on average 2-3 heterozygous subjects upon testing 20-30 individuals. To strike a balance between these two extremes, we therefore recommend genotyping at least 20-30 individual samples together with pooled DNA samples with the same whole-genome genotyping platform.

## Conclusions

UPDG integrates the functionalities of various programming environments (Unix/Linux shell, Perl and R) and provides the users with a one-stop service for pooled DNA GWAS analysis. Up to now, there are very few resources available for pooled DNA GWAS analysis, especially for Illumina platform. Existing resources require intense prior knowledge on programming and statistics. It is impossible to do pooled DNA GWAS analysis manually and hence use of computer programs is definitely required to achieve this. With limited resources available, scientists will be hindered and become reluctant to carry out pooled DNA GWAS despite being a well-established strategy for cost reduction. Different programming environments have their own edges and drawbacks. It is usually difficult to stick with one single programming language in complicated tasks like pooled GWAS analysis, but learning many programming languages at the same time is not feasible for most users. It is our intention to contribute our UPDG utilities package to the field of pooled DNA GWAS so that this useful research strategy will no longer be intractable to scientists with less advanced programming knowledge. UPDG provides users with an alternative choice of utilities packages for manipulating and analyzing their pooled DNA GWAS data. More importantly, it is our vision that the useful strategy of pooled DNA GWAS can gain in popularity by reducing the hindrance to data manipulation and analysis.

## Availability and Requirements

UPDG package for data analysis of pooled DNA GWAS (package components including Perl programs and R scripts, an example dataset and a user manual) is freely available (Additional file 1).

Project name: UPDG - Utilities package for data analysis of pooled DNA GWAS

Operating system: Platform independent

Programming language: Unix/Linux shell, Perl and R

Other requirements: ActivePerl, and Cygwin under Windows environment

License: GNU GPL v3

Any restriction to use by non-academics: On request and citation

## List of Abbreviations

ANOVA: Analysis of variance; GWAS: Genomewide association study.

## Authors' contributions

DWHH conceived the study, participated in the design and implementation of UPDG, and drafted the manuscript. MKHY participated in the design. SPY participated in the design and drafted the manuscript. All authors read and approved the final manuscript.

## Supplementary Material

Additional file 1 **UPDG**. UPDG package for data analysis of pooled DNA GWAS (package components including Perl programs and R scripts, an example dataset and a user manual). Click here for file
