# Supplementary material for: UPDG: Utilities package for data analysis of Pooled DNA GWAS
Source: BMC Genet. 2012 Jan 17;13:1. doi: 10.1186/1471-2156-13-1 (PMC3293712; doi:10.1186/1471-2156-13-1)
Supplement: Additional file 1 — UPDG. UPDG package for data analysis of pooled DNA GWAS (package components including Perl programs and R scripts, an example dataset and a user manual). [file 1471-2156-13-1-S1.ZIP › UPDG-UserManual.doc]

**UPDG (Utilities package for data analysis of Pooled DNA GWAS)**

**User Manual**

Section I.	Preliminary preparation

Section II.	Suggested working procedures

Section III.	Simplified working procedures

Section IV.	Components of UPDG

Section V.	Input file formats

Authors:	Daniel WH Ho, Maurice KH Yap, Shea Ping Yip

The Hong Kong Polytechnic University

Contact:	htdwhho@polyu.edu.hk

Last update:	20 November 2011

**Section I.	Preliminary preparation**

**a) Installation of ActivePerl**

If users want to execute the Perl programs in Windows environment, it is necessary to install the ActivePerl free community edition package from ActiveState (<http://www.activestate.com/activeperl>). As of July 2011, the version is 5.12.4. It is recommended to choose the MSI file for more convenient installation. There is also an option on 32-bit or 64-bit version to choose with. If users are using a 32-bit Windows system, the right option is Windows (x86) platform. On the other hand, a 64-bit Windows system should choose Windows (64-bit, x64) platform. Following the instructions from the installer and selecting the default settings will allow users to install ActivePerl conveniently.

**b) Installation of Cygwin**

If users have not installed Unix/Linux system or want to execute shell scripts in Windows environment, it would be useful to install Cygwin. Cygwin is a Linux-like environment for Windows. Generally speaking, many users are feeling more comfortable to work in Windows environment. Go to the website of Cygwin (<http://www.cygwin.com/>), download the setup.exe file (version 1.7.9-1 as of July 2011) and install Cygwin with default settings.

**c) Installation of R**

In UPDG, there are some R scripts using aov function from R and hence it is necessary to install R. From the website of R (<http://www.r-project.org/>), users can select an appropriate mirror site of CRAN (Comprehensive R Archive Network). Then, users should select precompiled binary distributions for Windows. The default is 32-bit Windows platform, but users can also select 64-bit alternative. Finally, choose the base subdirectory and download the .exe executable file (version 2.13.1 as of July 2011) to install R using default settings.

**Section II. Suggested working procedures**

**Step 1.**

**Generating input files for merge.pl**

There are 2 input files for merge.pl: one containing genotype data and another containing intensity data, both from individual DNA GWAS. These 2 sets of data are usually saved into separate files (original GWAS data files) along with other data. Hence, these original GWAS data files cannot be read directly by merge.pl because the file format is not appropriate. Unix/Linux shell operations can be used to extract the required data columns from the original GWAS data files.

For example, the original GWAS data files containing genotype and intensity data are called ind_genotype.txt and ind_intensity.txt, respectively. For ind_genotype.txt, tab-delimited columns 1-4 contain the required information (SampleID, MarkerID, allele 1 for the called genotype and allele 2 for the called genotype) for the genotype data of those markers (rs number in the second column of the example data set) to be further analyzed. These data can be extracted to a new file (ind_genotype_rs.txt) with Unix/Linux shell operation:

cat ind_genotype.txt | awk '/\trs/ {print $1"\t"$2"\t"$3"\t"$4}' > ind_genotype_rs.txt

If users want to perform the shell operation in Windows environment using Cygwin, start Cygwin and type the above command. Users are reminded that the input file ind_genotype.txt must be copied to the default directory of Cygwin (C:\cygwin\home\... where “...” is the user account name) before executing the command.

Data columns can be extracted from ind_intensity.txt similarly. The required information (MarkerID, SampleID, raw intensity for allele 1 of the called genotype and raw intensity for allele 2 of the called genotype) for intensity data is in columns 1-4 for those markers (rs number in the first column of the example data set) to be further analyzed. These data can be extracted to a new file (ind_intensity_rs.txt) with Unix/Linux shell operation:

cat ind_intensity.txt | awk '/^rs/ {print $2"\t"$1"\t"$3"\t"$4}' > ind_intensity_rs.txt

Please be noted that $1 represents column 1 of the file and so forth while "^" represents the beginning of line and "\t" represents tab-spacing. In the example, data columns are extracted in the specific order for the example dataset e.g. $1 $2 $3 $4 or $2 $1 $3 $4. However, if the original order of data columns in the input file is not appropriate for input data format for merge.pl (please refer to the information in next step), data columns should be extracted in appropriate order e.g. $2 $3 $1 $4, etc.

Note: UPDG can perform data analysis on Affymetrix data provided that they have been transformed into the necessary data file format. Detailed information on necessary data files and their formats have been provided in section IV and V. In particular, intensity data from Affymetrix CEL files can first be extracted using such free packages as SNPMaP or using the Affymetrix power tools (APT) provided by Affymetrix. Once the necessary data files are prepared, data analysis can then be handled by UPDG using steps 4 to 8.

**Step 2.**

**Merging genotype and intensity data for individual DNA GWAS from separate files with merge.pl**

The input files for merge.pl are ind_genotype_rs.txt and ind_intensity_rs.txt. Both files contain 4 columns. The file formats are specified in section IV. These files have been generated in step 1 and the command prompt of Windows environment needs to be started (Start menu > type cmd > ENTER). Users are reminded to put the input files and merge.pl into the same directory and enter such directory in the command prompt. Users need to specify the correct path to the working directory containing the data files and Perl programs by:

cd ../..

where "../.." is the path to the working directory.

Then, users can execute merge.pl with the command:

perl merge.pl ind_genotype_rs.txt ind_intensity_rs.txt ind_input_rs.txt

In this example, the output file name is chosen to be ind_input_rs.txt. However, users can specify their own output file name. This output file will be used as one of the input files for adjustment.pl in step 4.

**Step 3.**

**Generating data files for adjustment.pl**

The first input file for adjustment.pl in step 4 is a file containing raw intensity data for pooled DNA GWAS. The original pooled DNA GWAS data file contains such raw intensity data, but the format is not readable by adjustment.pl. Therefore, Unix/Linux shell operations are used to extract the required data columns from the original GWAS data file. The data should be sorted according to MarkerID as this order is necessary for operations in later steps.

If the original GWAS data file is called pooled_GWAS.txt and the columns 1, 2, 7 and 8 contain the required information (SampleID, MarkerID, raw intensity for allele 1 and raw intensity for allele 2) for those markers (rs number as the second column of the pooled_GWAS.txt) to be further analyzed, the extracted and sorted pooled DNA GWAS raw intensity data can be saved to a new file (pooled_intensity_rs.txt) with a Unix/Linux shell operation:

cat pooled_GWAS.txt | awk '/\trs/ {print $1"\t"$2"\t"$7"\t"$8}' | sort -k 2 > pooled_intensity_rs.txt

Please be noted that column 2 is MarkerID, and the operation "sort -k 2" sorts the extracted data by MarkerID.

Another original GWAS data file contains the information about marker chromosomal location, physical position, polymorphism, etc. This file should be available along with GWAS data. If this file is called SNP_map.txt, data of markers with rs number can be extracted to a new file (SNP_map_rs.txt) as:

cat SNP_map.txt | awk '/\trs/ {print $0}' > SNP_map_rs.txt

A phenotype data file (phenotype.txt) must be provided to adjustment.pl in step 4. This file contains 4 columns which specify the genotyping batch, phenotype and DNA pool information on each pooled sample in the pooled DNA GWAS. The file format for phenotype.txt is specified in section IV. The SampleID refers to those that appear in pooled_intensity_rs.txt. For genotyping batch, if all the genotyping is done in a single batch or the information is unknown, simply enter "1" for all samples. Otherwise, users can specify the batch information for samples accordingly. For phenotype, users are reminded to use character notation "A" for affected and "U" for unaffected, instead of 1 and 0. If there are 6 DNA pools for the affected group and 6 DNA pools for the unaffected group, the PoolID should be specified with character notations e.g. AP1, AP2 ... AP6 and UP1, UP2 ... UP6 for affected and unaffected pools, respectively. For replicates of the same pool, they should have the same PoolID.

**Step 4.**

**Estimating allele frequencies for markers from DNA pools, performing adjustment to correct for allelic preferential amplification, and generating input files for QC.pl with adjustment.pl**

There are 4 input files for adjustment.pl. The first input file is pooled_intensity_rs.txt – the raw intensity data for pooled DNA GWAS –from step 3. The second input file is ind_input_rs.txt from step 2. The third input file is SNP_map_rs.txt from step 3. The fourth input file is phenotype.txt from step 3. Users are required to specify the name of the output e.g. pooled_GWAS. The command for adjustment.pl is:

perl adjustment.pl pooled_intenstiy_rs.txt ind_input_rs.txt SNP_map_rs.txt phenotype.txt pooled_GWAS

For autosomal markers, allele frequencies unadjusted or adjusted by methods of Hoogendoorn et al. and Meaburn et al. and normalized by Craig et al. are generated in separate files. All the output files are named as pooled_GWAS, but with different suffixes.

| Suffix | Description | Output file |
| --- | --- | --- |
| _H | Output file for allele frequencies adjusted by method of Hoogendoorn et al. | pooled_GWAS_H |
| _M | Output file for allele frequencies adjusted by method of Meaburn et al. | pooled_GWAS_M |
| _N | Output file for allele frequencies normalized by method of Craig et al. and adjusted by method of Meaburn et al. | pooled_GWAS_N |
| _unadjusted | Output file for unadjusted allele frequencies | pooled_GWAS_unadjusted |

These output files generated will be used as the input for QC.pl in step 5.

**Step 5.**

**Removing markers with call rate or minor allele frequency (MAF) below a user-specified threshold, and generating filtered input data files for nested ANOVA with QC.pl**

The aov function of R is used to carry out nested analysis of variance (nested ANOVA). Error happens if the number of non-missing data values in either case or control group is below 3. Besides, SNPs with low call rate or low MAF are prone to genotyping errors. As a result, the generated data files from step 4 will be fed into QC.pl. Users are required to define the total number of data values per marker, a threshold for the minimum number of non-missing data values in each group (e.g., 3) and a threshold for MAF (e.g. 0.01). For the example data set, the total number of data values per marker is 36 (= 6*2*3 with 6 DNA pools for both case and control groups, and 3 technical replicates for each DNA pool). Since there are 4 files generated in step 4, each of them needs to be filtered by QC.pl to generate the filtered input file for nested ANOVA in step 6. The command for this part is:

perl QC.pl pooled_GWAS_H pooled_GWAS_H_filtered 36 3 0.01

perl QC.pl pooled_GWAS_M pooled_GWAS_M_filtered 36 3 0.01

perl QC.pl pooled_GWAS_N pooled_GWAS_N_filtered 36 3 0.01

perl QC.pl pooled_GWAS_unadjusted pooled_GWAS_unadjusted_filtered 36 3 0.01

**Step 6.**

**Performing nested ANOVA utilizing aov function of R with nested_ANOVA_[H/M/N/U].r**

The aov function in R is used to perform nested ANOVA. As R has been installed in the preliminary preparation step, R console can be started by double-clicking the desktop shortcut icon. Then, users can check the current directory by typing:

getwd()

Users need to specify the correct path to the working directory containing the data files and R scripts by:

setwd(".../...")

where ".../..." is the path leading to the working directory. For easy path setting, users can create a new working directory under the default directory of R and copy all the necessary data files and R scripts to it and then specify the path to this newly created working directory. Alternatively, users can simply copy all the data files and R scripts to the default directory of R without further setting a path to the working directory. Once the correct working directory has been set, users can then type the commands:

source("nested_ANOVA_H.r")

source("nested_ANOVA_M.r")

source("nested_ANOVA_N.r")

source("nested_ANOVA_U.r")

Since each command invokes the analysis of one dataset (e.g., nested_ANOVA_H.r will perform nested ANOVA using data from pooled_DNA_GWAS_H_filtered), this process is computationally intensive if large number of markers are involved. Users are recommended to execute the commands one by one, instead of starting multiple R consoles to execute them simultaneously. There are 4 datasets generated by correction methods of Hoogendoorn et al., Meaburn et al., Craig et al.+Meaburn et al., and unadjusted estimation. Each dataset will be handled by a separate R script (nested_ANOVA_[H/M/N/U].r).

As specified in each of the R scripts, some parameters need to be set. Users can open the Scripts in an ordinary text editor (e.g., WordPad or Notepad), make the required changes, and then save the changes accordingly. Any changes to the parameters will amend the R scripts accordingly to obtain correct analysis results, and thus should be made BEFORE the execution of the above commands to carry out nested ANOVA.

In row 1 of the R script, the output file name of nested ANOVA result is specified ("nested_ANOVA_result_H.txt" for "nested_ANOVA_H.r"). In row 4 of the R script, y is the variable specifying the total number of data values for a marker. For example, if there are 6 DNA pools for both case and control groups and each DNA pool has 3 technical replicates, there will be 6*2*3=36 data values in total and 36 should be assigned to variable y in row 4 of the R script. Users should amend the number in row 4 of the R script according to real situation of their own dataset. In row 5 of the R script, "1:3" specifies the data file contains 3 markers; and users should amend this part if the number of markers for data file is different (if the number of markers is 1000, 1:3 should be changed to 1:1000). Similarly, since “36” is set to be the total number of data values for the example data set, this value is also used in rows 10 and 11 of the R script; and users should amend these parts if the total number of data values is different.

**Step 7.**

**Organizing results from nested ANOVA and extracting markers with p values below a user-specified threshold with output_format.pl**

After nested ANOVA in step 6, results will be generated as 4 separate files (one for each dataset) and they should be copied to the same directory of output_format.pl. To facilitate convenient comparison of results, the 4 result files from step 6 can be summarized by output_format.pl, which generates 2 files. Users can choose not to proceed with all the 4 correction methods (Hoogendoorn et al., Meaburn et al., Craig et al.+Meaburn et al., and unadjusted estimation) in steps 5 and 6, but can still perform this step (no need to provide the missing data files) to generate result summary. The first file (nested_ANOVA_result_summary) is an overall summary specifying the p values of nested ANOVA from the 4 datasets. The second file (nested_ANOVA_result_sig_[threshold]) is a summary of significant markers with p values below a user-specified threshold e.g. 0.05 in at least one of the datasets. The command for executing output_format.pl is:

perl output_format.pl SNP_map_rs.txt 0.05

**Step 8.**

**Generating summary information on estimated allele frequencies of markers with mean_Rx_statistics.pl**

Last but not least, users may want to extract the estimated allele frequency (Rx) information from the data. By using the Perl program mean_Rx_statistics.pl, users can obtain the mean estimated allele frequencies from case and control pools as well as the absolute value of the difference between them. This information is available for all the 4 datasets from correction methods of Hoogendoorn et al., Meaburn et al., Craig et al.+Meaburn et al., and unadjusted estimation. The input files are the 4 output files from step 5 and users can specify the output file name e.g. allele_frequency_summary. Since there are 6 DNA pools for both case and control groups and each DNA pool has 3 technical replicates, there will be 6*2*3=36 data values in total and hence the total number of data values per marker is 36. Again, user can choose not to proceed with all the 4 correction methods (Hoogendoorn et al., Meaburn et al., Craig et al.+Meaburn et al., and unadjusted estimation) in step 5, but can still perform this step (no need to provide the missing data files) to generate estimated allele frequency summary. The command for mean_Rx_statistics.pl is:

perl mean_Rx_statistics.pl SNP_map_rs.txt 36 allele_frequency_summary

**Section III. Simplified working procedures**

**Windows environment**

**Step 1.**

Before executing the shell and batch scripts, users are reminded to make any necessary changes to the parameters of the commands in those scripts using text editor. (Please refer to section II for detailed instructions on parameter setting. The shell and batch scripts actually contain the commands mentioned in section II.)

**Step 2.**

Users should put all the necessary input files together with the components of UPDG into the same directory under Cygwin. The input files are:

ind_genotype.txt

ind_intensity.txt

pooled_GWAS.txt

SNP_map.txt

phenotype.txt

**Step 3.**

Execute the shell script "windows_data_preparation.sh" using Cygwin.

bash windows_data_preparation.sh

**Step 4.**

Execute the windows batch script "windows_data_analysis.bat" by double-clicking the icon for the batch script.

**Step 5.**

Execute the windows batch script "windows_result_summarization.bat" by double-clicking the icon for the batch script.

**Linux environment**

**Step 1.**

Before executing the shell and batch scripts, users are reminded to make any necessary changes to the parameter of the command in those scripts using text editor. (Please refer to section II for detailed instructions on parameter setting. The shell and batch scripts actually contain the commands mentioned in section II.)

**Step 2.**

Users should put all the necessary input files together with the components of UPDG into the same directory under Cygwin. The input files are:

ind_genotype.txt

ind_intensity.txt

pooled_GWAS.txt

SNP_map.txt

phenotype.txt

**Step 3.**

Execute the shell script "linux_UPDG_run.sh".

bash linux_UPDG_run.sh

**Section IV.	Components of UPDG**

| Component | Input file* | Output file* |
| --- | --- | --- |
| merge.pl | ind_genotype_rs.txt, ind_intensity_rs.txt | ind_input_rs.txt |
| adjustment.pl | Pooled_intensity_rs.txt, ind_input_rs.txt,  SNP_map_rs.txt,  phenotype.txt | pooled_GWAS_H, pooled_GWAS_M,  pooled_GWAS_N,  pooled_GWAS_unadjusted |
| QC.pl | pooled_GWAS_H, pooled_GWAS_M,  pooled_GWAS_N,  pooled_GWAS_unadjusted | pooled_GWAS_H_filtered, pooled_GWAS_M_filtered,  pooled_GWAS_N_filtered,  pooled_GWAS_unadjusted_filtered |
| nested_ANOVA_H.r,  nested_ANOVA_M.r,  nested_ANOVA_N.r,  nested_ANOVA_unadjusted.r | **pooled_GWAS_H_filtered, pooled_GWAS_M_filtered,**  **pooled_GWAS_N_filtered,**  **pooled_GWAS_unadjusted_filtered** | nested_ANOVA_result_H.txt, nested_ANOVA_result_M.txt, nested_ANOVA_result_N.txt, nested_ANOVA_result_U.txt |
| output_format.pl | **nested_ANOVA_result_H.txt, nested_ANOVA_result_M.txt, nested_ANOVA_result_N.txt, nested_ANOVA_result_U.txt**  SNP_map_rs.txt | nested_ANOVA_result_summary  nested_ANOVA_result_sig_[threshold] |
| mean_Rx_statistics.pl | **pooled_GWAS_H_filtered, pooled_GWAS_M_filtered,**  **pooled_GWAS_N_filtered,**  **pooled_GWAS_unadjusted_filtered,**  SNP_map_rs.txt | allele_frequency_summary |

*	Files indicated in regular font must be specified in the command. Files indicated in **boldface** are automatically read by the command (i.e., users must not change the file names).

**Section V.	File formats (Tab-delimited text)**

**ind_genotype_rs.txt**

Column 1	SampleID

Column 2	MarkerID

Column 3	Allele 1 for the called genotype in individual DNA GWAS

Column 4	Allele 2 for the called genotype in individual DNA GWAS

**ind_intensity_rs.txt**

Column 1	SampleID

Column 2	MarkerID

Column 3	Raw intensity for allele 1 of the called genotype in individual DNA GWAS

Column 4	Raw intensity for allele 2 of the called genotype in individual DNA GWAS

**ind_input_rs.txt**

Column 1	SampleID

Column 2	MarkerID

Column 3	Allele 1 for the called genotype in individual DNA GWAS

Column 4	Allele 2 for the called genotype in individual DNA GWAS

Column 5	Raw intensity for allele 1 of the called genotype in individual DNA GWAS

Column 6	Raw intensity for allele 2 of the called genotype in individual DNA GWAS

**pooled_intensity_rs.txt**

Column 1	SampleID

Column 2	MarkerID

Column 3	Raw intensity for allele 1 in pooled DNA GWAS

Column 4	Raw intensity for allele 2 in pooled DNA GWAS

**SNP_map_rs.txt**

Column 1	Index

Column 2	MarkerID

Column 3	Chromosome

Column 4	Physical position

Column 5	GenTrain score

Column 6	SNP

Column 7	ILMN strand

Column 8	Customer strand

Column 9	NormID

**phenotype.txt**

Column 1	SampleID for pooled DNA sample

Column 2	Batch

Column 3	Phenotype

Column 4	PoolID

**pooled_GWAS_[H/M/N/unadjusted]**

Column 1	SampleID

Column 2	Batch

Column 3	Phenotype

Column 4	PoolID

Column 5	MarkerID

Column 6	Estimated allele frequency

Column 7	k correction factor (omitted in pooled_GWAS_unadjusted)

**pooled_GWAS_[H/M/N/unadjusted]_filtered**

Column 1	SampleID

Column 2	Batch

Column 3	Phenotype

Column 4	PoolID

Column 5	MarkerID

Column 6	Estimated allele frequency

Column 7	k correction factor (omitted in pooled_GWAS_unadjusted)

**nested_ANOVA_result_[H/M/N/U].txt**

Not formatted (generated by aov function of R)

**nested_ANOVA_result_summary**

Column 1	MarkerID

Column 2	Chromosome

Column 3	Physical position

Column 4	Nested ANOVA p value for unadjusted dataset

Column 5	Nested ANOVA p value for dataset adjusted using method of Hoogendoorn et al.

Column 6	Nested ANOVA p value for dataset adjusted using method of Meaburn et al.

Column 7	Nested ANOVA p value for dataset adjusted using method of Craig et al.+Meaburn et al.

**nested_ANOVA_result_sig_[threshold]**

Column 1	MarkerID

Column 2	Chromosome

Column 3	Physical position

Column 4	Nested ANOVA p value for unadjusted dataset

Column 5	Nested ANOVA p value for dataset adjusted using method of Hoogendoorn et al.

Column 6	Nested ANOVA p value for dataset adjusted using method of Meaburn et al.

Column 7	Nested ANOVA p value for dataset adjusted using method of Craig et al.+Meaburn et al.

**allele_frequency_summary**

Column 1	MarkerID

Column 2	# of case pools

Column 3	# of control pools

Column 4	Mean case pool estimated allele frequency (unadjusted)

Column 5	Mean control pool estimated allele frequency (unadjusted)

Column 6	Absolute difference between Mean case and control pool estimated allele frequency	(unadjusted)

Column 7	Mean case pool estimated allele frequency (method of Hoogendoorn et al.)

Column 8	Mean control pool estimated allele frequency (method of Hoogendoorn et al.)

Column 9	Absolute difference between Mean case and control pool estimated allele frequency	(method of Hoogendoorn et al.)

Column 10	Mean case pool estimated allele frequency (Meaburn et al.)

Column 11	Mean control pool estimated allele frequency (Meaburn et al.)

Column 12	Absolute difference between Mean case and control pool estimated allele frequency	(Meaburn et al.)

Column 13	Mean case pool estimated allele frequency (Craig et al.+Meaburn et al.)

Column 14	Mean control pool estimated allele frequency (Craig et al.+Meaburn et al.)

Column 15	Absolute difference between Mean case and control pool estimated allele frequency	(Craig et al.+Meaburn et al.)
